# Defining and benchmarking open problems in single-cell analysis

**DOI:** 10.21203/rs.3.rs-4181617/v1

**Published:** 2024-04-04

**Authors:** Malte D. Luecken, Scott Gigante, Daniel B. Burkhardt, Robrecht Cannoodt, Daniel C. Strobl, Nikolay S. Markov, Luke Zappia, Giovanni Palla, Wesley Lewis, Daniel Dimitrov, Michael E. Vinyard, D.S. Magruder, Alma Andersson, Emma Dann, Qian Qin, Dominik J. Otto, Michal Klein, Olga Borisovna Botvinnik, Louise Deconinck, Kai Waldrant, The Open Problems Jamboree Members, Jonathan M. Bloom, Angela Oliveira Pisco, Julio Saez-Rodriguez, Drausin Wulsin, Luca Pinello, Yvan Saeys, Fabian J Theis, Smita Krishnaswamy

**Affiliations:** 1 Institute of computational Biology, Helmholtz Munich, Neuherberg, Germany; 2 Institute of Lung Health & Immunity, Helmholtz Munich; Member of the German Center for Lung Research (DZL), Munich, Germany.; 3 Immunai, New York, USA; 4 Cellarity, Inc. Somerville, USA; 5 Data Intuitive, Lebbeke, Belgium; 6 Data Mining and Modelling for Biomedicine group, VIB Center for Inflammation Research, Ghent, Belgium; 7 Department of Applied Mathematics, Computer Science, and Statistics, Ghent University, Ghent, Belgium; 8 Institute of Clinical Chemistry and Pathobiochemistry, School of Medicine, Technical University of Munich, Munich, Germany; 9 TUM School of Life Sciences Weihenstephan, Technical University of Munich, Germany; 10 Division of Pulmonary and Critical Care Medicine, Feinberg School of Medicine, Northwestern University; 11 Department of Mathematics, School of Computing, Information and Technology, Technical University of Munich, Munich, Germany; 12 Interdepartmental Program in Computational Biology and Bioinformatics, Yale University, New Haven, CT 06511, USA; 13 Heidelberg University, Faculty of Medicine, and Heidelberg University Hospital, Institute for Computational Biomedicine, Heidelberg, Germany; 14 Department of Chemistry and Chemical Biology, Harvard University, Cambridge, MA, USA; 15 Broad Institute of Harvard and MIT, Cambridge, MA, USA; 16 Molecular Pathology Unit, Center for Cancer Research, Massachusetts General Hospital, Boston, MA, USA; 17 Department of Computer Science, Yale University, New Haven CT, USA; 18 Genentech Inc; 19 Royal Institute of Technology (KTH), Gene Technology; 20 Science for Life Laboratory (SciLifeLab); 21 Wellcome Sanger Institute, Wellcome Genome Campus, Cambridge, UK; 22 Basic Sciences Division, Fred Hutchinson Cancer Center, Seattle WA; 23 Computational Biology Program, Public Health Sciences Division, Seattle WA; 24 Translational Data Science IRC, Fred Hutchinson Cancer Center, Seattle WA; 25 Apple; 26 Data Sciences Platform, Chan Zuckerberg Biohub, 499 Illinois St, San Francisco, CA 94158; 27 Bridge Bio Pharma, 3160 Porter Drive, Suite 250, Palo Alto, CA, 94304; 28 Massachusetts Institute of Technology; 29 Insitro, South San Francisco; 30 VIB Center for AI & Computational Biology (VIB.AI), Gent, Belgium; 31 Cellular Genetics Programme, Wellcome Sanger Institute, Hinxton, UK (associated faculty); 32 Department of Genetics, Yale University, New Haven CT, USA

## Abstract

With the growing number of single-cell analysis tools, benchmarks are increasingly important to guide analysis and method development. However, a lack of standardisation and extensibility in current benchmarks limits their usability, longevity, and relevance to the community. We present Open Problems, a living, extensible, community-guided benchmarking platform including 10 current single-cell tasks that we envision will raise standards for the selection, evaluation, and development of methods in single-cell analysis.

Single-cell genomics has enabled the study of biological processes at an unprecedented scale and resolution^1–3^. These studies were enabled by innovative data generation technologies coupled with emerging computational tools specialised for single-cell data. As single-cell technologies have become more prevalent, so has the development of new analysis tools, which have resulted in over 1700 published algorithms^4^ (as of February 2024). Thus, there is an increasing need to continuously evaluate which algorithm performs best in which context to inform best practices^5,6^ that evolve with the field.

In many fields of quantitative science, public competitions and benchmarks address this need by evaluating state-of-the-art methods against known criteria, following the concept of a Common Task Framework^7^. Public competitions of this kind have a rich track record of accelerating innovation in algorithm development in computer vision (ImageNet^8^), natural language processing (GLUE^9^), robotics (RoboCup^10^), recommendation systems (Netflix Challenge^11^), and, more recently in the life sciences, in protein structure prediction (CASP^12^) or systems biology (DREAM^13^).

In single-cell genomics, as in many other domains, it is typical for analysis algorithms to be evaluated via benchmarks. However, such benchmarks are often of limited use as the field suffers from a lack of standardised procedures for benchmarking^14^, which can lead to different assessments of the same method. Bespoke benchmarks set up by method developers to evaluate their own algorithms often include datasets and metrics chosen to highlight the advantage of their tools, which has been shown to lead to less objective assessments^15,16^. Alternatively, independent benchmarks that evaluate the current state of the art in a given area^17–20^ may be less biased, but their results are static and inevitably age. These frameworks are also typically not designed for extensibility or interoperability, limiting the value of reusing a framework to perform new systematic benchmarks^14^. This inability to reuse infrastructure leads to repeats of non-standardized benchmarks that cannot provide the guidance that users need. For example, at least four benchmarks of batch integration methods exist^18,21–23^, each of which uses different sets of datasets and metrics and thus suggest different optimal methods (Fig 1a). Similar issues have been reported across single-cell topics, where datasets and metrics typically have less than 10% overlap between benchmarks^24^. Finally, even if standard benchmarks are defined, historical analysis has shown that decentralised implementation of such benchmarks tends to inflate model performance due to custom hyperparameter selection and data processing^25^. Standardised benchmarking that guides users and promotes method innovation can only be achieved by neutral, independent efforts with ongoing community participation^14,15,24^. Such community participation around quantified tasks requires continuous updates, a process that is hard to realise in the typical result-paper framework that defines the modern scientific process.

To achieve this goal, we developed the Open Problems in Single-Cell Analysis (Open Problems) platform. The Open Problems platform is an open-source, extensible, living benchmarking framework that enables quantitative evaluation of best practices in single-cell analysis. It combines a permissively licensed GitHub repository (github.com/openproblems-bio/openproblems) with community-defined tasks, an automated benchmarking workflow, and a website to explore the results. Currently, Open Problems includes 10 defined tasks, on which 16 datasets are used to evaluate 70 methods using 31 metrics. These tasks were defined by community engagement, including on the public GitHub repository, in regular open meetings, and at a hackathon in March 2021 with over 50 participants. This broad involvement has already led to new benchmarking insights and best practice recommendations, while improving and standardizing previously published benchmarks. We envision that Open Problems’ community-defined standards for progress in single-cell data science will raise the bar for the selection and evaluation of methods, provide targets for novel method innovation, and enable developers without single-cell expertise to contribute to the field.

To enable truly living benchmarks, we designed a standardised and automated infrastructure that allows members of the single-cell community to contribute to Open Problems in a seamless manner (Methods). Each Open Problems task consists of datasets, methods, and metrics (Fig 1b). Datasets define both the input and the ground truth for a task, methods attempt to solve the task, and metrics evaluate the success of a method on a given dataset. We provide cloud infrastructure to enable centralised benchmarking when new methods, datasets, or metrics are added to our platform. Within each task, every method is evaluated on every dataset using every metric, and each method is then ranked on a per-dataset basis by the average normalised metric score and presented in a summary table on the Open Problems website (https://openproblems.bio).

Community engagement on the platform is centered around an open discussion forum, open code contribution opportunities, and task leadership. Task leaders are community members who have contributed substantially to a task, assume organisational responsibilities for the task, and are ultimately responsible for task definition, maintenance, and facilitation of community contributions. Task definitions, choices of metrics, and implementations of methods are discussed on our GitHub repository and can be easily amended by pull requests which are reviewed by task leaders and the core infrastructure team.

To enable seamless community involvement in Open Problems, we have designed our platform to leverage cloud infrastructure that provides reproducibility, accessibility, and automation (**Supplementary Figure 1**). Each task is organised as a directory with subdirectories for datasets, methods, metrics, and utilities. Each task must contain at least one dataset, one metric, and two baseline methods, which provide upper and lower bounds for performance of the task. Each component (i.e. dataset, method, or metric) exists as a single file in the relevant subdirectory, and adding a new method is as simple as opening a pull request to the repository and adding a new file that follows the API for that task. When a community member adds a component, the new contribution is automatically tested in the cloud. When all tests pass and the new contribution is accepted, the results from the new contribution are automatically submitted to the Open Problems website. To maximise reproducibility, each component is run within a Docker container defined by the method contributor, and all data is downloaded from public repositories, including figshare, the Gene Expression Omnibus (GEO)^26^, and CELLxGENE^27^.

Building on previous work defining open challenges in single-cell analysis^28^ and independent benchmarking studies in single-cell genomics^18,19,21,29–36^, we started by defining seven Open Problems tasks (Fig. 2a), which extends to 10 with the inclusion of subtasks. While several tasks were directly informed by published benchmarking papers (e.g., batch correction^18^, cell-cell communication^37^), others were defined by method developers in the single-cell community (e.g., spatial decomposition). These tasks are designed to be a starting point on which further community development can be added to address further open problems.

A typical task setup can be exemplified by the cell-cell communication (CCC) task (Fig 2b; **Supplementary Note 2.1)**. The goal of cell-cell communication inference methods is to infer which cell types are communicating within a tissue to mediate tissue function. Typical algorithms base predictions on the expression of ligand and receptor genes in dissociated single-cell data^38^. Ground-truth data for cellular communication are challenging to obtain. Thus, this task is divided into two subtasks that use different proxies for this ground truth: spatial colocalization (source-target subtask) and cytokine activity (ligand-target subtask). As the CCC methods included in this task^39–42^ typically score ligand-receptor pairs using either their expression magnitude or cell-type specificity, *mean* and *max* aggregation functions are used to score interaction strengths between source and target cell types (source-target task) or ligands and target cell types (ligand-target task)^39–42^. The outputs of these methods are finally evaluated using the area under the precision-recall curve and odds ratios. These metrics measure how well ground truth source-target (co-localized cell types) or ligand-target (cytokine activity within a cell type) pairs are prioritised when ranking all interactions and how many true pairs are found in the top 5%, respectively.

While the CCC task was contributed to Open Problems on the basis of a published benchmark^37^, the task definition and metrics evolved based on input from the community and the Open Problems team. This process has enabled the Open Problems results to generate insight beyond the initial publication (Fig 2c), which focused predominantly on the comparison of CCC databases and showed variable method performance across tasks. In the CCC Open Problems task, we find that methods that rely on expression magnitude outperform approaches that rely on expression specificity. Indeed, the top performers across tasks are CellPhoneDB and LIANA’s ensemble model of expression magnitude scoring methods. Furthermore, *max* aggregation of ligand-receptor scores outperformed *mean* aggregation across tasks and methods. This improved inference of cellular communication using only the top-predicted interactions suggests that methods are better at prioritising a small fraction of relevant interactions while being prone to noise when their full interaction rankings are considered. Thus, analysts interpreting CCC results may likewise want to focus only on the most high-scoring predictions when inferring which cell types interact (**Supplementary Note 1.1**).

Using this combination of expert knowledge with community input, we also provide best-practice recommendations for preprocessing and method selection for label projection, dimensionality reduction for 2D visualisation, batch integration, spatial decomposition, denoising, and matching cellular profiles across modalities (**Supplementary Note 1**). For example, on all four reference datasets currently included in the Open Problems label projection task, a simple logistic regression model outperforms more complex methods that explicitly model batch effects, such as Seurat^43^ or scANVI^44^, even when noise is added to the training data (**Supplementary Note 1.2**). Moreover, we also show that it is easier to correct for batch effects in single-cell graphs compared to in latent embeddings or expression matrices (**Supplementary Note 1.4**), and denoising methods perform best with non-standard preprocessing approaches that better stabilise variance (**Supplementary Note 1.6**). Overall, Open Problems tasks provide best-practice recommendations to data analysts that can be continuously updated and thereby increase in robustness as new methods are developed and more complex datasets become available.

Open Problems living benchmarking tasks also function as a quantifiable target for the development of new methods. This problem definition is particularly useful for the wider machine learning community that may lack domain knowledge (i.e. single-cell expertise). Leveraging the batch integration and matching modality tasks as a basis, we previously set up popular competitions for multimodal data integration at NeurIPS 2021^45,46^ and 2022, with over 260 and 1,600 participants, respectively. In these competitions, the developers of multiple top performers had no prior experience with single-cell data, yet were able to submit solutions that substantially outperform state-of-the-art methods^45^. We envision that the Open Problems platform will drive method development by improving the accessibility of open challenges in single-cell analysis via defined tasks. To promote this, Open Problems enables method developers to submit both prototype and final solutions to the platform for automated evaluation against the current state-of-the-art. Open Problems results, which are made available under a Creative Commons Attribution licence (CC-BY), can then be included in the respective method papers. Similarly, entirely new benchmarks can be implemented as tasks, run via Open Problems, and published separately while remaining updatable.

Taken together, the Open Problems platform is a community resource that quantitatively defines open challenges in single-cell analysis, determines the current state-of-the-art solutions, promotes method development to improve on these solutions, and monitors progress towards these goals. Open Problems addresses the issues of bespoke and decentralised benchmarking by providing standardised but flexible infrastructure and task definitions. Thereby, Open Problems enables broader accessibility for scientists to contribute to the advancement of the field of single-cell analysis. We envision Open Problems to bring about a shift in perspective on method selection for data analysts and method evaluation for developers, supporting a transition towards higher standards for methods in single-cell data science.

## Online Methods

### Infrastructure

The Open Problems infrastructure is designed considering three core principles: automation, reproducibility, and ease of contribution. Where possible, all steps involved in the integration of new contributions to the living benchmark are automated with minimal manual review. All of the components involved in generating the benchmark are publicly accessible and documented, and contributing guides are made available to ensure that all community members are able to contribute to the benchmark. Briefly, the Open Problems infrastructure consists of two GitHub repositories that orchestrate continuous integration and continuous deployment via GitHub Actions workflows, using Nextflow^47^, Nextflow Tower and AWS to run the benchmark, and Quarto and Netlify^48^ to render and host the website (**Fig S1**).

### Code structure

**Figure 1:**
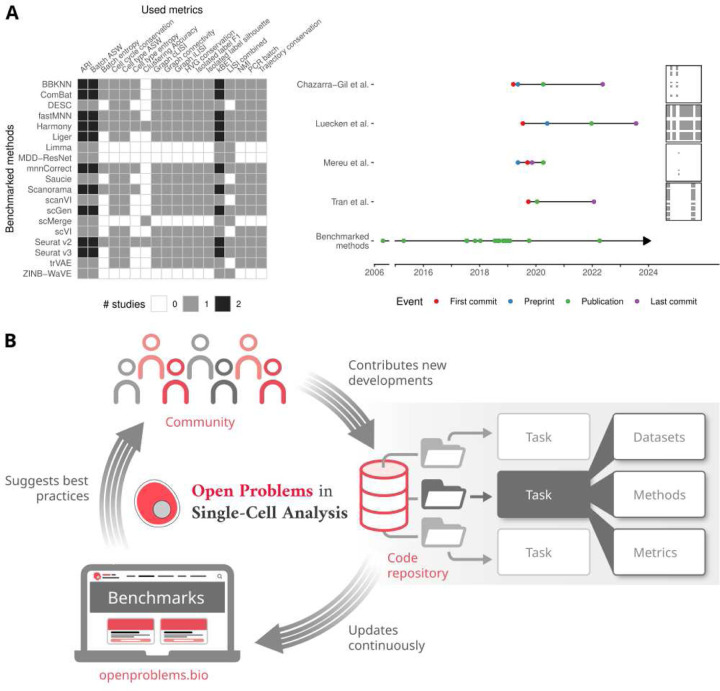
The Open Problems in Single-cell Analysis living benchmarking platform. A) Overview and timeline of published benchmarks of single-cell batch integration. Four publications have benchmarked 19 methods using 18 metrics. Light grey and black squares indicate whether one or two benchmarks include this method-metric combination (left). Arrows indicate the range of publication times of methods included in the benchmark. B) Schematic diagram of the Open Problems platform. The Open Problems platform consists of tasks that are broken down into datasets, methods, and metrics. The community contributes code to these tasks in the platform, which uses these contributions to extend the benchmarks that are run and pushed to the Open Problems website. The community can then consult the website for guidance on method selection.

Each task in the benchmark is broken down into three core components: datasets, methods, and metrics. Datasets provide a single-cell dataset with known ground truth corresponding to the task, methods perform the task, and metrics evaluate the methods’ performance with respect to the defined task (Fig 1b). Each time the living benchmark is updated, every method is run on all datasets and evaluated using all metrics in the task to give the final score presented on the website.

Datasets, methods, and metrics are written as single Python functions, which are executed inside a Docker container to ensure that all external dependencies can be made available for a given method. Datasets return an AnnData object,^49^ methods accept this AnnData object and return a modified AnnData object, and metrics accept the modified AnnData object and return a floating-point value. In order to encourage contributions from the community, Open Problems also provides a simple wrapper function to execute R code via scprep^50^ and rpy2^51^ in order to avoid limiting developers to a single programming language. Additionally, the Open Problems repository also provides a number of utility functions used across multiple tasks. These include data loaders, which download publicly available data that may be used as datasets in multiple tasks, normalisers, which provide standardised approaches to normalising raw data, and Docker images, which provide common sets of dependencies used across many datasets, methods, and metrics.

### Metric normalization

Metrics can have different effective ranges when evaluating methods for a particular task. While these different ranges may not affect method comparisons using only one metric, they do affect benchmarking results when multiple performance metric scores must be combined to give an overall ranking of methods. In order to equalise the contribution of each metric to the final score, we use a system of “baseline methods”, which are designed to approximate both optimal and random performance on a given task for each metric. Since metrics in a task may be optimised by different baseline methods, we consider the optimum score of a given metric as the maximum score achieved by any baseline method and random performance as the minimum score achieved by any baseline method. All method scores are then normalised to this range such that optimum performance corresponds to a normalised score of 1 and random performance to a normalised score of 0. Following best practices for machine learning competitions^52^, each method’s score is then averaged over all normalised scores to give the method’s overall score. Note that for some metrics (e.g., R squared in a regression task), it is possible to perform arbitrarily worse than random. In this case, methods may achieve scores significantly less than 0.

### Benchmark procedure

The Open Problems living benchmark is run periodically on all contributions via Nextflow Tower (https://tower.nf). When a new release is created in GitHub, the benchmark is triggered via a Tower Actions webhook, which directs Nextflow Tower to launch a Nextflow^47^ pipeline. This pipeline generates all datasets, runs all methods on each dataset, and computes all metrics on each method-dataset pair for each task. The results of this benchmark are metric scores and compute resources used for each method on each dataset. The computation for this pipeline is run on AWS Batch and stored on AWS S3. Following the successful completion of a benchmark run, Nextflow Tower triggers a GitHub webhook to download the results from S3, process them, and commit them to the Open Problems website repository, which displays these results on the website.

### Continuous Integration

To ensure community contributions to Open Problems function as intended, we implement a series of automated tests applied to all contributions to the repository. Unit tests, implemented with PyTest and run on GitHub Actions, ensure that all tasks, datasets, methods, and metrics conform to the expected API. This is achieved through a combination of universal- and task-specific API checks, which confirm that each function produces the intended output defined in each task. Additionally, each task must define a sample dataset and method, which are used as input for testing the implementation of methods and metrics respectively. Datasets and methods are also expected to respond to a *test* keyword, which requires that the returned dataset be made smaller for testing purposes and the runtime of long methods be curtailed (e.g. by reducing the number of iterations) to ensure unit testing can be completed with minimal computational resources. Once all unit tests successfully pass, the full benchmark is run (with the test flag) to ensure that all dataset-method-metric combinations are compatible. Finally, in order to merge contributions to the main branch of the repository, test coverage is checked with Codecov (https://codecov.io/) to ensure that all new code is covered by the unit tests, and a manual review by a code maintainer is required to ensure the contributed code follows community standards, including the Open Problems Code of Conduct located at https://github.com/openproblems-bio/openproblems/blob/main/CODE_OF_CONDUCT.md.

### Continuous Deployment

The information on the Open Problems website (https://openproblems.bio) is composed of a) static content stored in the website repository, b) metadata stored in the Open Problems code repository, and c) results data from the latest benchmarking run. Each time the metadata or results data are updated, a pull request is automatically created via GitHub Actions to commit these changes to the website repository. Changes to the website repository are rendered with Quarto (https://quarto.org/) and hosted by Netlify^48^.

### Development

Ease of contribution by the community is one of the central design principles for the Open Problems infrastructure. To facilitate the contribution of new methods and optimization of existing methods, we provide a command-line interface (CLI) to Open Problems. This CLI enables developers to locally evaluate the results of their contributions in a targeted manner (i.e. running only the submitted method rather than the full benchmark) and without prior experience with the Open Problems repository. The CLI provides a simple one-line command to load any dataset, run any method (given a dataset), or compute a metric (given the output of a method). Additionally, a detailed guide for contributing datasets, methods, metrics, and new tasks is maintained at https://github.com/openproblems-bio/openproblems/blob/main/CONTRIBUTING.md.

### Open Problems tasks

Open Problems tasks are classified as stub or full tasks to denote task maturity. Stubs consist of at least one dataset, three methods, and one metric while tasks are regarded as full once they encompass at least two datasets, six methods, and a metric. Tasks that do not qualify as stubs are regarded as “*under discussion*” and are omitted here. This classification serves to communicate to users at which point meaningful guidance can be derived from the results of an Open Problems benchmark. Here we outline the setup of currently defined tasks in the Open Problems platform, encompassing six full tasks (including five subtasks) and one stub task. Details on datasets, methods, and metrics, as well as discussion of task results and interpretation, are elaborated on in **Supplementary Note 1**.

### Cell-cell communication

To harmonise the different tools and resources, we used the LIANA framework as a foundation for the cell-cell communication task^37^. To generate a ground truth for CCC benchmarking, we used alternative data modalities that provide insight into cellular communication such as spatial proximity and cytokine signalling. Each modality corresponds to a subtask. In the source-target subtask, we assess whether putatively interacting cell types are close to each other in spatial data. In the ligand-target subtask, downstream cytokine activities are used to infer whether a cytokine ligand was indeed active within a target cell type.

### Label projection

To benchmark label projection methods, each dataset is divided into reference and query subsets. Methods are trained on the reference data subset and predict cell type labels for the query data subset. This prediction is evaluated against the true labels to quantify method performance. Different train and test splits are evaluated on several datasets.

### Dimensionality reduction for 2D visualisation

The dimensionality reduction task attempts to quantify the ability of methods to embed the information present in complex single-cell studies into a two-dimensional space. Thus, this task is specifically designed for dimensionality reduction for visualisation and does not consider other uses of dimensionality reduction in standard single-cell workflows, such as improving the signal-to-noise ratio (and in fact, several of the methods use PCA as a pre-processing step for this reason). Unlike most tasks, methods for the dimensionality reduction task must accept a matrix containing expression values normalised to 10,000 counts per cell and log transformed (log-10k) and produce a two-dimensional coordinate for each cell. Pre-normalised matrices are required in order to enforce consistency between the metric evaluation (which generally requires normalised data) and the method runs. When these are not consistent, methods that use the same normalisation as used in the metric tend to score more highly. For some methods, we also evaluate the pre-processing recommended by the method.

### Batch integration

In this task, we evaluate batch integration methods on their ability to remove batch effects in the data while conserving variation attributed to biological effects. As methods that integrate batches can output three different data formats (feature matrices, embeddings and/or neighbourhood graphs), we split the batch integration task into three subtasks. As input, all tasks take a combined normalised dataset with multiple batches and consistent cell-type labels. The respective batch-integrated representation (matrix, embedding, or graph) is then evaluated using sets of metrics that capture how well batch effects are removed and whether biological variance is conserved. We have based this particular task on a recent, extensive benchmark of single-cell data integration methods^18^.

### Spatial decomposition

The spatial decomposition task revolves around inferring relative cell type abundances in array-based spatial transcriptomics data. Specifically, the task requires methods to estimate the composition of cell identities (i.e., cell type or state) that are present at each capture location (i.e., spot or bead). The cell identity estimates are presented as proportion values, representing the proportion of the cells at each capture location that belong to a given cell identity. The faithfulness of this inference is evaluated using several metrics. In this task, we distinguish between reference-based decomposition and de novo decomposition, where the former leverages external data (e.g., scRNA-seq or scNuc-seq) to guide the inference process, while the latter only works with the spatial data. In this task, it is required that all datasets have an associated reference single-cell data set to perform reference-based decomposition, but methods are free to ignore this information to perform de novo decomposition instead. All methods benchmarked so far require a scRNA-seq reference to learn the cell-type-specific transcriptomics signature.

### Denoising

Single-cell RNA-sequencing data can be notoriously noisy, with molecular capture rates that often hover around 40% for droplet-based sequencing^53^ and up to 95% of measured zeros^54^. To address this noise, data augmentations that denoise or “impute” scRNA-seq expression matrices have been proposed. The data denoising task attempts to evaluate the major data denoising tools and to implement reasonable and universal metrics across a variety of datasets. The methods that are considered take as input a scRNA-seq expression matrix, which is then randomly partitioned into “train” and “test” subsets using the molecular cross validation (MCV) approach^55^. MCV creates train and test splits by simulating two random samples from the observed reads in each cell of the dataset. Once the training set has been denoised, its similarity to the testing set is assessed via one of several loss functions. Although datasets are assumed to already contain only the cells and genes that pass initial pre-processing steps, further normalisation is considered a part of the evaluated method. To facilitate the comparison of model performance using MCV, each denoising method is applied to the “train” subset, and model outputs are evaluated against the “test” subset using various metrics.

### Matching modalities

In this stub task, the goal is to learn a latent space where cells profiled by different technologies in different modalities are matched if they have the same state. We use jointly profiled data as ground truth so that we can evaluate when the observations from the same cell acquired using different modalities are similar. A perfect result has each of the paired observations sharing the same coordinates in the latent space. A method that can achieve this would be able to match datasets across modalities to enable multimodal cellular analysis from separately measured profiles.

## Figures and Tables

**Figure 2:**
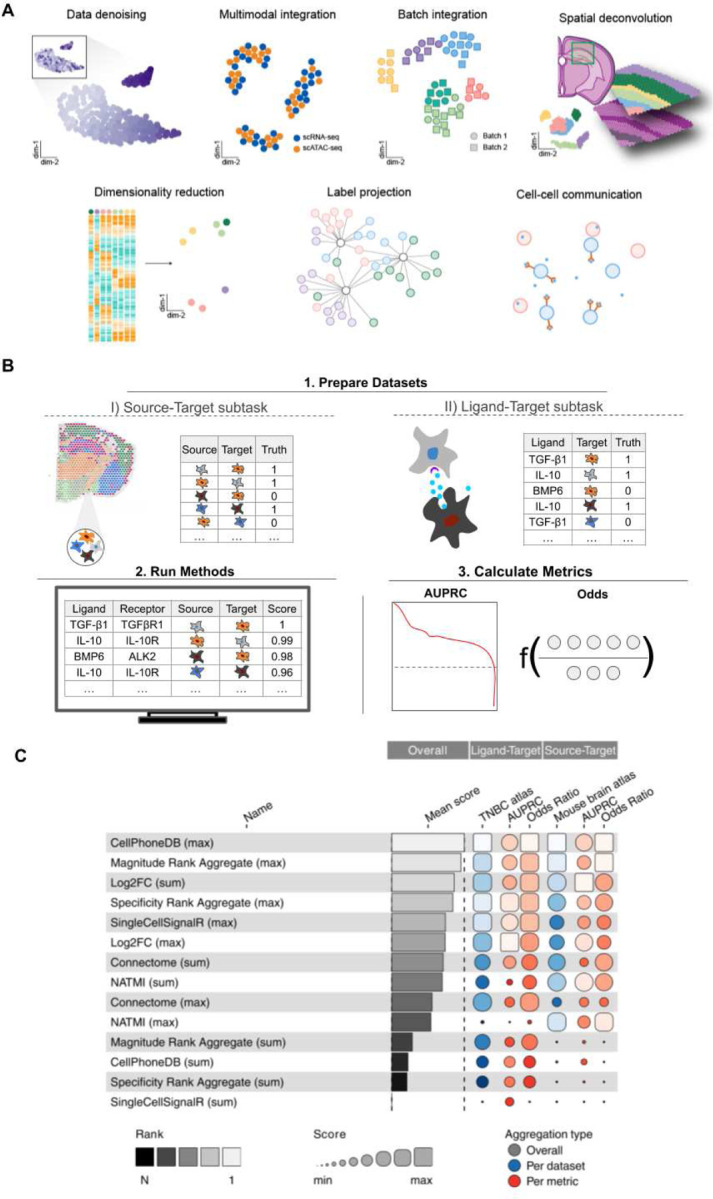
Task overview, setup and results. A) Overview of the seven tasks currently included in the Open Problems platform. Batch integration and cell-cell communication (CCC) consist of three and two subtasks respectively, making up the current total of 10 tasks. B) Schematic diagram of the CCC task. This task includes two subtasks defined by different types of ground truth: spatial cell type co-localization in the source-target subtask and cytokine profiling in the ligand-target subtask. Methods are run on each subtask to score the likelihood of interaction between source and target cell types or ligand and target cell types. Finally, the area under the precision-recall curve (AUPRC) and the odds ratio of true to false positive interactions in the top 5% of predicted pairs are used to score method outputs (**Supplementary Note 1.1**). C) Collated results of both CCC subtasks. Methods are ranked using the mean of the overall score for each subtask (shown as “TNBC Atlas” and “Mouse brain atlas” blue boxes respectively). These overall scores are computed as the mean of all scaled metric results (red boxes). Linear scaling is performed using random and perfect baseline methods, whose performance is set to 0 and 1, respectively (see Methods).

## Data Availability

All Open Problems code is publicly available at https://www.github.com/openproblems-bio/openproblems. This code includes data loaders for all datasets used with associated metadata on where this data came from. Code to reproduce the figures is publicly available at https://github.com/openproblems-bio/nbt2023-manuscript/. Furthermore, detailed information on all datasets are available at https://openproblems.bio/datasets/.

## References

[1] Cao J. A human cell atlas of fetal gene expression. Science 370, (2020). 10.1126/science.aba7721 PMC7780123 33184181

[2] Montoro D. T. A revised airway epithelial hierarchy includes CFTR-expressing ionocytes. Nature 560, 319–324 (2018). 30069044 10.1038/s41586-018-0393-7 PMC6295155

[3] Plass M. Cell type atlas and lineage tree of a whole complex animal by single-cell transcriptomics. Science 360, eaaq1723 (2018). 29674432 10.1126/science.aaq1723

[4] Zappia L., Phipson B. & Oshlack A. Exploring the single-cell RNA-seq analysis landscape with the scRNA-tools database. PLoS Comput. Biol. 14, e1006245 (2018). 29939984 10.1371/journal.pcbi.1006245 PMC6034903

[5] Heumos L. Best practices for single-cell analysis across modalities. Nat. Rev. Genet. (2023). 10.1038/s41576-023-00586-w PMC10066026 37002403

[6] Luecken M. D. & Theis F. J. Current best practices in single-cell RNA-seq analysis: a tutorial. Mol. Syst. Biol. 15, e8746 (2019). 31217225 10.15252/msb.20188746 PMC6582955

[7] Donoho D. 50 Years of Data Science. Journal of Computational and Graphical Statistics vol. 26 745–766 Preprint at 10.1080/10618600.2017.1384734 (2017).

[8] Deng J. Imagenet: A large-scale hierarchical image database. in 2009 IEEE conference on computer vision and pattern recognition 248–255 (Ieee, 2009).

[9] Wang A. GLUE: A Multi-Task Benchmark and Analysis Platform for Natural Language Understanding. arXiv [cs.CL] (2018).

[10] Kitano H. RoboCup-97: Robot Soccer World Cup I. (Springer Science & Business Media, 1998).

[11] Lanning J. B. S. & Bennett J. Netflix Prize. Proc. KDD Cup and Workshop 2007.

[12] Moult J. A decade of CASP: progress, bottlenecks and prognosis in protein structure prediction. Curr. Opin. Struct. Biol. 15, 285–289 (2005). 15939584 10.1016/j.sbi.2005.05.011

[13] Meyer P. & Saez-Rodriguez J. Advances in systems biology modeling: 10 years of crowdsourcing DREAM challenges. Cell Syst 12, 636–653 (2021). 34139170 10.1016/j.cels.2021.05.015

[14] Sonrel A. Meta-analysis of (single-cell method) benchmarks reveals the need for extensibility and interoperability. Genome Biol. 24, 119 (2023). 37198712 10.1186/s13059-023-02962-5 PMC10189979

[15] Brooks T. G., Lahens N. F., Mrčela A. & Grant G. R. Challenges and best practices in omics benchmarking. Nat. Rev. Genet. (2024) doi:10.1038/s41576-023-00679-6. 38216661

[16] Buchka S., Hapfelmeier A., Gardner P. P., Wilson R. & Boulesteix A.-L. On the optimistic performance evaluation of newly introduced bioinformatic methods. Genome Biol. 22, 152 (2021). 33975646 10.1186/s13059-021-02365-4 PMC8111726

[17] Saelens W., Cannoodt R., Todorov H. & Saeys Y. A comparison of single-cell trajectory inference methods. Nat. Biotechnol. 37, 547–554 (2019). 30936559 10.1038/s41587-019-0071-9

[18] Luecken M. D. Benchmarking atlas-level data integration in single-cell genomics. Nat. Methods 19, 41–50 (2022). 34949812 10.1038/s41592-021-01336-8 PMC8748196

[19] Soneson C. & Robinson M. D. Bias, robustness and scalability in single-cell differential expression analysis. Nat. Methods 15, 255–261 (2018). 29481549 10.1038/nmeth.4612

[20] Squair J. W. Confronting false discoveries in single-cell differential expression. Nat. Commun. 12, 5692 (2021). 34584091 10.1038/s41467-021-25960-2 PMC8479118

[21] Chazarra-Gil R., van Dongen S., Kiselev V. Y. & Hemberg M. Flexible comparison of batch correction methods for single-cell RNA-seq using BatchBench. Nucleic Acids Res. 49, e42 (2021). 33524142 10.1093/nar/gkab004 PMC8053088

[22] Tran H. T. N. A benchmark of batch-effect correction methods for single-cell RNA sequencing data. Genome Biol. 21, 12 (2020). 31948481 10.1186/s13059-019-1850-9 PMC6964114

[23] Mereu E. Benchmarking single-cell RNA-sequencing protocols for cell atlas projects. Nat. Biotechnol. 38, 747–755 (2020). 32518403 10.1038/s41587-020-0469-4

[24] Cao Y. The current landscape and emerging challenges of benchmarking single-cell methods. bioRxiv 2023.12.19.572303 (2023) doi:10.1101/2023.12.19.572303. PMC12495992 41045508

[25] Musgrave K., Belongie S. & Lim S.-N. A Metric Learning Reality Check. arXiv [cs.CV] (2020).

[26] Edgar R., Domrachev M. & Lash A. E. Gene Expression Omnibus: NCBI gene expression and hybridization array data repository. Nucleic Acids Res. 30, 207–210 (2002). 11752295 10.1093/nar/30.1.207 PMC99122

[27] Megill C. cellxgene: a performant, scalable exploration platform for high dimensional sparse matrices. bioRxiv 2021.04.05.438318 (2021) doi:10.1101/2021.04.05.438318.

[28] Lähnemann D. Eleven grand challenges in single-cell data science. Genome Biol. 21, 31 (2020). 32033589 10.1186/s13059-020-1926-6 PMC7007675

[29] Li B. Benchmarking spatial and single-cell transcriptomics integration methods for transcript distribution prediction and cell type deconvolution. Nat. Methods 19, 662–670 (2022). 35577954 10.1038/s41592-022-01480-9

[30] Hou W., Ji Z., Ji H. & Hicks S. C. A systematic evaluation of single-cell RNA-sequencing imputation methods. Genome Biol. 21, 218 (2020). 32854757 10.1186/s13059-020-02132-x PMC7450705

[31] Raimundo F., Vallot C. & Vert J.-P. Tuning parameters of dimensionality reduction methods for single-cell RNA-seq analysis. Genome Biol. 21, 212 (2020). 32831127 10.1186/s13059-020-02128-7 PMC7444048

[32] Sun X., Lin X., Li Z. & Wu H. A comprehensive comparison of supervised and unsupervised methods for cell type identification in single-cell RNA-seq. Brief. Bioinform. 23, (2022). 10.1093/bib/bbab567 PMC8921620 35021202

[33] Sun S., Zhu J., Ma Y. & Zhou X. Accuracy, robustness and scalability of dimensionality reduction methods for single-cell RNA-seq analysis. Genome Biol. 20, 269 (2019). 31823809 10.1186/s13059-019-1898-6 PMC6902413

[34] Huang Y. & Zhang P. Evaluation of machine learning approaches for cell-type identification from single-cell transcriptomics data. Brief. Bioinform. 22, (2021). 10.1093/bib/bbab035 33611343

[35] Avila Cobos F., Alquicira-Hernandez J., Powell J. E., Mestdagh P. & De Preter K. Benchmarking of cell type deconvolution pipelines for transcriptomics data. Nat. Commun. 11, 5650 (2020). 33159064 10.1038/s41467-020-19015-1 PMC7648640

[36] Cantini L. Benchmarking joint multi-omics dimensionality reduction approaches for the study of cancer. Nat. Commun. 12, 124 (2021). 33402734 10.1038/s41467-020-20430-7 PMC7785750

[37] Dimitrov D. Comparison of methods and resources for cell-cell communication inference from single-cell RNA-Seq data. Nat. Commun. 13, 3224 (2022). 35680885 10.1038/s41467-022-30755-0 PMC9184522

[38] Armingol E., Baghdassarian H. M. & Lewis N. E. The diversification of methods for studying cell–cell interactions and communication. Nat. Rev. Genet. 1–20 (2024). 38238518 10.1038/s41576-023-00685-8 PMC11139546

[39] Efremova M., Vento-Tormo M., Teichmann S. A. & Vento-Tormo R. CellPhoneDB: inferring cell-cell communication from combined expression of multi-subunit ligand-receptor complexes. Nat. Protoc. 15, 1484–1506 (2020). 32103204 10.1038/s41596-020-0292-x

[40] Hou R., Denisenko E., Ong H. T., Ramilowski J. A. & Forrest A. R. R. Predicting cell-to-cell communication networks using NATMI. Nat. Commun. 11, 5011 (2020). 33024107 10.1038/s41467-020-18873-z PMC7538930

[41] Raredon M. S. B. Computation and visualization of cell-cell signaling topologies in single-cell systems data using Connectome. Sci. Rep. 12, 4187 (2022). 35264704 10.1038/s41598-022-07959-x PMC8906120

[42] Cabello-Aguilar S. SingleCellSignalR: inference of intercellular networks from single-cell transcriptomics. Nucleic Acids Res. 48, e55 (2020). 32196115 10.1093/nar/gkaa183 PMC7261168

[43] Stuart T. Comprehensive Integration of Single-Cell Data. Cell 177, 1888–1902.e21 (2019). 31178118 10.1016/j.cell.2019.05.031 PMC6687398

[44] Xu C. Probabilistic harmonization and annotation of single-cell transcriptomics data with deep generative models. Mol. Syst. Biol. 17, e9620 (2021). 33491336 10.15252/msb.20209620 PMC7829634

[45] Lance C. Multimodal single cell data integration challenge: results and lessons learned. in Proceedings of the NeurIPS 2021 Competitions and Demonstrations Track 162–176 (2022).

[46] Luecken M. D. A sandbox for prediction and integration of DNA, RNA, and proteins in single cells. in Proceedings of the Neural Information Processing Systems Track on Datasets and Benchmarks 1 (NeurIPS Datasets and Benchmarks 2021) (2021).

[47] Di Tommaso P. Nextflow enables reproducible computational workflows. Nat. Biotechnol. 35, 316–319 (2017). 28398311 10.1038/nbt.3820

[48] Attardi J. Using Gatsby and Netlify CMS. (Apress).

[49] Virshup I., Rybakov S., Theis F. J., Angerer P. & Alexander Wolf F. anndata: Annotated data. bioRxiv 2021.12.16.473007 (2021) doi:10.1101/2021.12.16.473007.

[50] Burkhardt D. B. Quantifying the effect of experimental perturbations at single-cell resolution. Nat. Biotechnol. 39, 619–629 (2021). 33558698 10.1038/s41587-020-00803-5 PMC8122059

[51] Gautier L. An intuitive Python interface for Bioconductor libraries demonstrates the utility of language translators. BMC Bioinformatics 11 Suppl 12, S11 (2010). 10.1186/1471-2105-11-S12-S11 PMC3040525 21210978

[52] Maier-Hein L. Why rankings of biomedical image analysis competitions should be interpreted with care. Nat. Commun. 9, 5217 (2018). 30523263 10.1038/s41467-018-07619-7 PMC6284017

[53] Hagemann-Jensen M. Single-cell RNA counting at allele and isoform resolution using Smart-seq3. Nat. Biotechnol. 38, 708–714 (2020). 32518404 10.1038/s41587-020-0497-0

[54] Hicks S. C., Townes F. W., Teng M. & Irizarry R. A. Missing data and technical variability in single-cell RNA-sequencing experiments. Biostatistics 19, 562–578 (2018). 29121214 10.1093/biostatistics/kxx053 PMC6215955

[55] Batson J., Royer L. & Webber J. Molecular Cross-Validation for Single-Cell RNA-seq. bioRxiv 786269 (2019) doi:10.1101/786269.

